# Autonomic synchrony induced by hyperscanning interoception during interpersonal synchronization tasks

**DOI:** 10.3389/fnhum.2023.1200750

**Published:** 2023-07-21

**Authors:** Michela Balconi, Roberta A. Allegretta, Laura Angioletti

**Affiliations:** ^1^International Research Center for Cognitive Applied Neuroscience (IrcCAN), Università Cattolica del Sacro Cuore, Milan, Italy; ^2^Research Unit in Affective and Social Neuroscience, Department of Psychology, Università Cattolica del Sacro Cuore, Milan, Italy

**Keywords:** social interoception, autonomic synchrony, interpersonal physiology, physiological synchrony, hyperscanning

## Abstract

According to previous research, people influence each other’s emotional states during social interactions via resonance mechanisms and coordinated autonomic rhythms. However, no previous studies tested if the manipulation of the interoceptive focus (focused attention on the breath for a given time interval) in hyperscanning during synchronized tasks may have an impact on autonomic synchrony. Thus, this study aims to assess the psychophysiological synchrony through autonomic measures recording during dyadic linguistic and motor synchronization tasks performed in two distinct interoceptive conditions: the focus and no focus on the breath condition. 26 participants coupled in 13 dyads were recruited. Individuals’ autonomic measures [electrodermal: skin conductance level and response (SCL, SCR); cardiovascular indices: heart rate (HR) and HR variability (HRV)] was continuously monitored during the experiment and correlational coefficients were computed to analyze dyads physiological synchrony. Inter-subject analysis revealed higher synchrony for HR, HRV, SCL, and SCR values in the focus compared to no focus condition during the motor synchronization task and in general more for motor than linguistic task. Higher synchrony was also found for HR, SCL, and SCR values during focus than no focus condition in linguistic task. Overall, evidence suggests that the manipulation of the interoceptive focus has an impact on the autonomic synchrony during distinct synchronization tasks and for different autonomic measures. Such findings encourage the use of hyperscanning paradigms to assess the effect of breath awareness practices on autonomic synchrony in ecological and real-time conditions involving synchronization.

## 1. Introduction

Does paying attention to one’s body signals while performing a synchronization task affect the autonomic system synchrony between two interagents?

Literature demonstrated that, during social exchanges, individuals modify one other’s states and behaviors through basic resonance mechanisms (Golland et al., 2015). Indeed, sharing other people’s emotional states provides the interagents an embodied framework for comprehending their intents and behaviors, enabling them to not only comprehend other individuals’ intentions but also to sync with them (Niedenthal, 2007; Keysers et al., 2010; Balconi and Bortolotti, 2013; Balconi and Canavesio, 2016). Recent studies showed that such synchronization might develops during social exchanges in the form of an alignment of behavior (Richardson et al., 2007; Konvalinka et al., 2010), posture (Shockley et al., 2003), neurophysiological (Dumas et al., 2011; Hasson et al., 2012) as well as physiological measurements (McFarland, 2001; Müller and Lindenberger, 2011; Smith et al., 2011).

To grasp the complexity of such synchronization and to deepen the interpersonal dynamics between two individuals, the employment of an “hyperscanning” paradigm in neuroscience allowed the shifting from a single-subject approach to a “two person neuroscience” (Schilbach, 2010) and enabled the simultaneous recording of the cortical activity from two or more participants interacting together (Montague et al., 2002) by creating spatiotemporal maps of cerebral regions involved in the generation of social interactions (Babiloni and Astolfi, 2014; Balconi and Vanutelli, 2016).

By exploiting this paradigm, former research in the field of interpersonal autonomic physiology investigated the autonomic synchrony during joint tasks in populations with a specific relational bond (e.g., parent-child, mother-infant, couples, teammates, psychotherapist-client, and others) and how this physiological synchrony can be influenced by distinct variables (Palumbo et al., 2017). For instance, Chatel-Goldman et al. (2014) found that interpersonal touch increased coupling of electrodermal activity between the interacting partners. Also, we have shown that during cooperative social interactions, Skin Conductance Level and Response (SCL and SCR) indices, together with heart rate (HR), increased in contexts with high emotional engagement, where the cooperative motivation was induced by presenting feedback which reinforced the positive outcomes of the intersubjective exchange (Balconi and Bortolotti, 2012; Vanutelli et al., 2017a,^2018^; Balconi et al., 2019).

However, to the best of our knowledge, no previous research explored the impact of the manipulation of the attention to one’s body signals on autonomic synchrony while dyads are performing synchronization tasks. The attention to one’s body signal for a given time interval is an interoceptive dimension known as Interoceptive Attentiveness (IA; Schulz, 2016), that has been formerly operationalized as the attention on spontaneous breath and it was shown to influence autonomic reactivity in the context of empathy for pain (Angioletti and Balconi, 2021).

With reference to breath, previous research showed a synchronization between individuals’ respiratory rate (such as those of dancers and audience members) during a shared condition (Bachrach et al., 2015). More interestingly, a recent study showed that client alliance and the therapist assessment of the progress of a therapeutic session positively correlate with the physiological synchrony between clients and psychotherapists (Tschacher and Meier, 2020). These works suggested that participants’ breathing rate synchrony can occur in relation to positive interactive dynamics. It remains to be clarified whether the simple attention to the breath could have an impact on autonomic synchrony during even basic synchronization tasks.

Among the experimental paradigms used to reproduce social dynamics, motor and linguistic synchronization tasks have often been employed in previous neural and physiological hyperscanning works (Balconi and Vanutelli, 2017; Palumbo et al., 2017; Kelsen et al., 2022) and also in this study, two simple motor and speech synchronization tasks, which consist of modified versions of the finger tapping task (Jobbágy et al., 2005) and the alternate speech task (Kawasaki et al., 2013), respectively, were selected.

By using such basic synchronization tasks, previous hyperscanning studies performed in the field of social interoception (the field of studies investigating the relation between interoception and social processes) demonstrated that the interoceptive attention on spontaneous breath might impact interpersonal neural synchronization during social dynamics.

Indeed, through an electrophysiological (EEG) hyperscanning approach, we explored the EEG markers of interpersonal tuning of neurotypical participants during simple dyadic synchronization tasks (motor- and cognitive-based) performed in two distinct interoceptive conditions, that is when the attention of the participants was focused on their breath versus not focused on their breath (Balconi and Angioletti, 2023b). Results showed greater EEG coherence for alpha band in frontopolar brain regions and in central brain regions within the dyads, during the focus on the breath condition for the motor compared to the cognitive synchronization task; during the same experimental condition, delta and theta band showed augmented inter-individual coherence in frontal region and central areas. Also, Coomans et al. (2021) observed inter-subject EEG coherence (for theta and alpha bands) while healthy dyads were practicing an exercise requiring paying attention of the breath (i.e., a mindful breathing exercise performed without controlling the respiratory rate or synchrony).

Additionally, in a recent functional Near Infrared Spectroscopy (fNIRS) hyperscanning study, it was observed a significantly higher inter subject hemodynamic coherence in the left prefrontal cortex (PFC) when dyads performed both the synchronization tasks with a social compared to no-social frame and concurrently focused their attention on IA (Balconi and Angioletti, 2023b). The work of Balconi and Angioletti (2023b) added to the previous evidence the proof that the interoceptive focus, together with the presence of a social frame may favor the manifestation of a left PFC interpersonal tuning during synchronization tasks.

Taken together, this evidence suggests that the attention to breathing shared between two individuals leads to interpersonal neural synchrony during dyadic interactions.

By moving toward a two-person neuroscience approach and with a specific focus on the physiological level, this study aimed at testing the effect of the explicit IA manipulation (operationalized as the focus of the breath) on autonomic synchrony during synchronization tasks. The computation of coherence indices adopted in former studies on cooperative and competitive joint actions (Balconi and Vanutelli, 2018), real-life conversations in the work context, such as a performance interview (Venturella et al., 2017; Balconi et al., 2020) and a job assessment interview (Balconi and Cassioli, 2022; Balconi et al., 2022), will be here exploited explore the physiological synchrony.

Considering the results from the previous EEG hyperscanning study (Balconi and Angioletti, 2023b), we hypothesized to observe higher coherence in autonomic indices during the focus on the breath condition mainly in the motor compared to the linguistic synchronization task, since the motor synchronization task was previously shown to be more sensitive to the interoceptive manipulation (Angioletti and Balconi, 2022b).

Secondly, during the focus on the breath condition in the motor compared to the linguistic synchronization task, we expected to observe this inter-subject coherence effect for EDA indices, as markers of higher shared emotional engagement (Vanutelli et al., 2017b), and cardiovascular indices, with increased HR coherence as index of togetherness (Noy et al., 2015), and higher HR variability coherence as index of synchrony (Vickhoff et al., 2013) between the individuals.

Finally, considering that inter-subject coherence indices were previously adopted in psychophysiological studies to explore autonomic synchrony, we aim to test if they can be considered as a valid marker of physiological synchronization, when the interoceptive focus is manipulated.

## 2. Materials and methods

### 2.1. Study sample

With the use of a non-probabilistic convenience sampling strategy, a total of 26 university students were recruited for the current experiment (16 females; age mean = 25.41; standard deviation = 0.12) and were matched in 13 dyads. No power analysis was conducted in the absence of a population that could serve as a reference sample. Each dyad consisted of two participants of the same sex who were age-matched and had never met before the trial. All participants were with right-handedness and had normal or corrected-to-normal vision. Pregnancy, previous contemplative experience, severe physical and chronic illnesses, convulsions, chronic pain, and any mental or neurological abnormalities were among the criteria for exclusion. After being informed they would not be compensated for their participation, they voluntarily joined the study and completed written informed consent forms. The Ethics Committee of the Department of Psychology (Catholic University of the Sacred Heart in Milan, Italy) gave its approval for this study (2020 TD-a.a.2020–2021), which was conducted in conformity with the new version of the Declaration of Helsinki (2013).

### 2.2. Synchronization tasks description

In the current investigation, two synchronization tasks - simple motor and language synchronization tasks - were adopted. During the whole course of the experiment, each component of the dyad was allowed to see the other component.

For the motor synchronization tasks, the subjects had to synchronize and coordinate their finger-tapping actions for 3 min with their partner as part of the motor synchronization task. The participants were instructed to position the fingers of their dominant hand about a centimeter apart while sitting in a chair with their elbows resting on a table. They were told to tap the table using all their dominant hand fingers. They did not need to move at a certain speed or to spread their fingers as far as they could. They had to replicate the finger movements of the partner. The finger-tapping task was performed for about 60 times.

In the linguistic synchronization task, the subjects had to syllabicate in unison with their partner for 3 min as part of a modified version of the human-to-human alternating speech task. The four syllables “LA,” “BA,” “CA,” and “DA” had to be spoken alternately and in that order by the participants. For example, when one member of the dyad uttered “CA,” the second member should have paired the syllable by saying “CA” to pronounce a syllable at the same time. There was no pre-selection of speech patterns. There were at least 45 repetitions from “LA” to “DA” in each loop during the 3 min. These tasks were employed also in prior fNIRS and EEG hyperscanning research and were adopted in this experimental study to maintain consistency (Angioletti and Balconi, 2022a; Balconi and Angioletti, 2023a).

### 2.3. Procedure and experimental manipulation

Before the experiment, participants were given procedural instructions on how to complete the two synchronization tasks under different experimental conditions that manipulated IA. The participants were instructed to regulate IA in the first condition by concentrating on their breathing: “*We ask you to focus on your breathing as you complete this task. While you do the exercise, try to pay attention to how you’re feeling and whether your breathing changes.*” The subjects were not instructed to breathe at a specific rate. In contrast, no particular instructions were given to participants in the control condition (which did not involve any modification of interoception) and they were just required to finish the tasks. The task execution was randomized and counterbalanced for the type of the task and the condition to avoid order effect.

**FIGURE 1:**
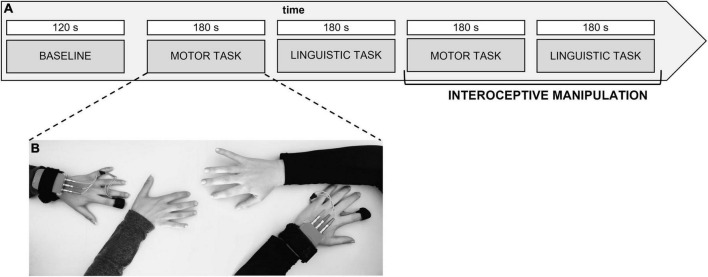
**(A,B)** Experimental procedure. Experimental procedure describing the setting for the synchronization task and the autonomic data acquisition from the dyad. The task execution was randomized and counterbalanced for the type of the task and the condition to avoid order effect.

The manipulation of IA was tested during the debriefing phase following the experiment. Participants evaluated the amount of attention to their breathing on a Visual Analogue Scale from 0 to 10, as well as their perception of synchrony. *“From 0 to 10, how much attention did you focus to yourself throughout the task?”* was the question asked to the participants to gauge their level of self-awareness. In the focus condition, all participants’ average scores on the tasks were above 5 points (*M* = 8.75; SD = 1.08), whereas in the no focus condition, average scores were lower (*M* = 5.97; SD = 1.54). The whole experimental procedure lasted about 45 min (Figures 1A, B).

### 2.4. Autonomic data recording

The autonomic activity was gathered and recorded using two X-pert2000 portable Biofeedback system with a MULTI radio module (Schuhfried GmbH, Modling, Austria) that allows monitoring SCR in lS, SCL in lS, and HR in bpm (bpm). A peripheral sensor was applied to the second finger of the non-dominant hand’s distal phalanx to record data. The SCL value was recorded using a current-current measurement using an EDA gold electrode and a sampling frequency of 2 kiloHertz (kHz). Alternating voltage was used to lessen polarization. The SCL has a measurement resolution of 12 nanoseconds (nS) and was collected at a sampling frequency of 20 Hz. HR was collected using photoplethysmography at a sampling frequency of 500 Hertz (Hz). The non-dominant hand was tracked using a transmitting unit’s accelerometer in meter/square second (m/s2) to avoid hand movements from interfering with the recordings. Both standard measures of cardiac activity [HR, inter-beat interval (IBI)] and a measure of HR variability (the standard deviation of IBI of normal sinus beats, SDNN) were computed after the inspection of qualitative and quantitative data to identify and remove recording (motor) or biological artifacts in order to have a broad picture of stress-related cardiac responses as well as a measure of vagal tone, which is connected to the functionality of parasympathetic recovery mechanisms that foster the return to baseline (Mendes, 2009). Trials with motor artifacts were removed from the analyses. A baseline activity was recorded for 120 s before the tasks began. After artifact rejection, autonomic activity collected at rest and during the tasks was segmented and averaged across conditions to calculate mean condition-specific SCL, SCR, HR, and HRV modulations via an *ad hoc* automated VBA script designed to localize event-markers and calculate condition-specific metrics.

### 2.5. Data analysis

#### 2.5.1. Coherence value analysis

The partial correlation coefficient Πij for each dyad, was computed in a first analysis to determine the indexes coherence. These indices were created by normalizing the inverse of the covariance matrix:

Γ = (Γ_*ij*_) = Σ^−1^: inverse of the covariance matrix
$\Pi_{i\,j}=-\frac{\Gamma_{i\,j}}{\sqrt{\Gamma_{i\,i}\,\Gamma_{j\,j}}}$ : partial correlation matrix

This methodology allows for the evaluation of two signals (i, j) that are not related to one another, and it was frequently used in earlier neurophysiological studies (Balconi and Angioletti, 2022, 2023b).

#### 2.5.2. Statistical analysis

The coherence values were subjected to a second round of analysis, where coefficients were treated as the dependent measures of a repeated measures ANOVA with independent within-factors of condition (2: focus, no focus) and task (2: motor, cognitive). Any significant interactions between simple effects were explored using pairwise comparisons for all ANOVA tests, and the Bonferroni correction was used to reduce the potential bias of repeated comparisons. All ANOVA tests’ degrees of freedom were modified as needed using the Greenhouse-Geisser epsilon. The magnitudes of the statistically significant effects were determined using partial eta squared (η_*p*_^2^) indices.

## 3. Results

A description of two sets of results that correlate to the two analyses done on the autonomic dependent measures will be described in the following paragraphs. Coherence analysis was used for each dyad in the investigation as a first step. Subsequently, we implemented an inferential statistical ANOVA test to the coherence values regarded as dependent measures.

### 3.1. First set of results: inter-subject coherence results

**FIGURE 2:**
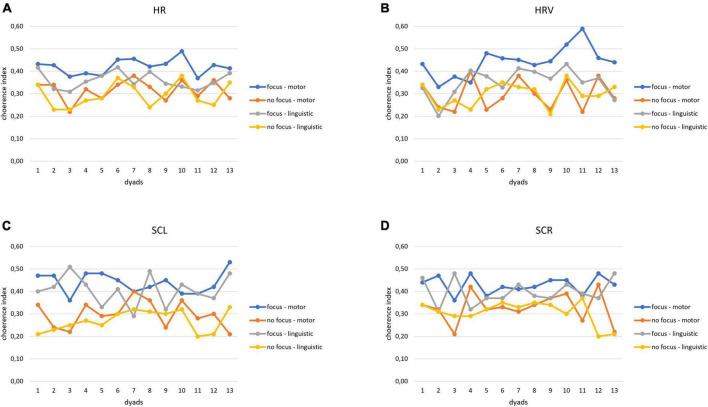
**(A–D)** Psychophysiological coherence indices for autonomic data. Trend of the coherence indices modulation as a function of the synchronization tasks for HR **(A)**, HRV **(B)**, SCL **(C),** and SCR **(D)** in each dyad.

For the first step of analysis, we found the computed coherence values for each index (SCR, SCL, HR, and HRV) in each experimental condition. In the graphs below, we have reported for this first step the mean trend of the coherence index for each dyad of participants (Figures 2A–D).

### 3.2. Second set of results: ANOVA results

The ANOVAs applied to the inter-subject coherence indices as dependent variables for each dyad revealed significant effects for the autonomic indices. The following paragraphs report the significant results obtained for the ANOVAs.

#### 3.2.1. HR

**FIGURE 3:**
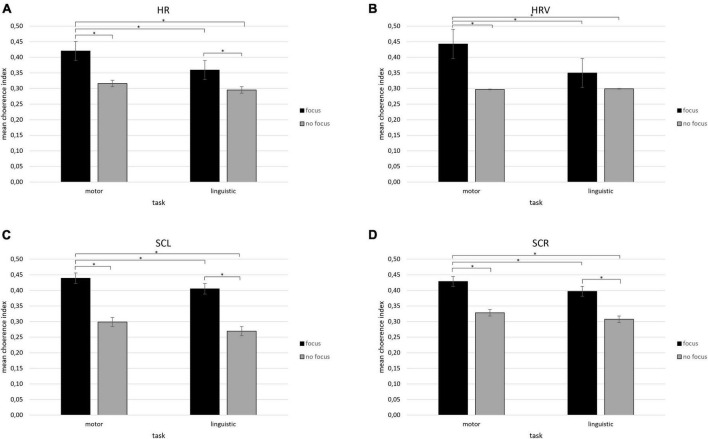
**(A–D)** Mean coherence indices for HR, HRV, SCL, and SCR. Bar graphs show the mean values of coherence indices (± SE) for **(A,B)** cardiovascular indices (HR, HRV) and **(C,D)** electrodermal (SCL, SCR) indices under the focus condition during the motor compared to the linguistic synchronization task. All asterisks (*) mark statistically significant differences, with *p* ≤ 0.01.

A first significant interaction effect was observed for Condition × Task [*F*(1,12) = 9.45, *p* ≤ 0.01, η_*p*_^2^ = 0.452]. Pairwise comparisons showed an increase in coherence of HR values in the focus condition when participants performed the motor task compared to the focus condition while participants performed the linguistic task [*F*(1,12) = 8.34, *p* ≤ 0.01, η_*p*_^2^ = 0.39] and compared to the no focus condition for both tasks {motor [*F*(1,12) = 7.76, *p* ≤ 0.01, η_*p*_^2^ = 0.37] and linguistic [*F*(1,12) = 8.14, *p* ≤ 0.01, η_*p*_^2^ = 0.38]}. Moreover, an increase in coherence of HR values was found in the focus condition compared to the no focus condition while participants performed the linguistic task [*F*(1,12) = 9.06, *p* ≤ 0.01, η_*p*_^2^ = 0.411] (Figure 3A). No other significant effects were found for the HR index.

#### 3.2.2. HRV

For HRV, it was detected a significant interaction effect for Condition × Task [*F*(1,12) = 9.34, *p* ≤ 0.01, η_*p*_^2^ = 0.41]. Pairwise comparison revealed that HRV values increased in coherence in the focus condition when participants carried out the motor task compared to the focus condition while subjects performed the linguistic task [*F*(1,12) = 7.43, *p* ≤ 0.01, η_*p*_^2^ = 0.38] and compared to the no focus condition for both tasks {motor [*F*(1,12) = 8.45, *p* ≤ 0.01, η_*p*_^2^ = 0.40] and linguistic [*F*(1,12) = 0.94, *p* ≤ 0.01, η_*p*_^2^ = 0.43]} (Figure 3B). No other significant effects were found.

#### 3.2.3. SCL and SCR

About SCL, a significant interaction effect was observed for Condition × Task [*F*(1,12) = 8.56, *p* ≤ 0.01, η_*p*_^2^ = 0.38]. An increase in coherence of SCL values was found by pairwise comparisons in the focus condition when participants performed the motor task compared to the focus condition while participants performed the linguistic task [*F*(1,12) = 7.04, *p* ≤ 0.01, η_*p*_^2^ = 0.35] and compared to the no focus condition for both tasks {motor [*F*(1,12) = 7.30, *p* ≤ 0.01, η_*p*_^2^ = 0.35] and linguistic [*F*(1,12) = 6.78, *p* ≤ 0.01, η_*p*_^2^ = 0.32]}. Furthermore, greater SCL coherence values were found in the focus compared to the no focus condition when participants executed the linguistic task [*F*(1,12) = 8.04, *p* ≤ 0.01, η_*p*_^2^ = 0.39] (Figure 3C). No other significant effects were found for the SCL index.

Finally, for SCR, a significant Condition × Task interaction effect was found [*F*(1,12) = 8.90, *p* ≤ 0.01, η_*p*_^2^ = 0.40]. Greater coherence of SCR values in the focus condition when participants performed the motor task compared to the focus condition while participants carried out the linguistic task [*F*(1,12) = 7.76, *p* ≤ 0.01, η_*p*_^2^ = 0.37] and compared to the no focus condition for both tasks (motor [*F*(1,12) = 7.04, *p* ≤ 0.01, η_*p*_^2^ = 0.38] and linguistic [*F*(1,12) = 6.34, *p* ≤ 0.01, η_*p*_^2^ = 0.32] were shown by pairwise comparisons. Moreover, higher SCR coherence was detected in the focus condition compared to the no focus condition while participants performed the linguistic task [*F*(1,12) = 8.01, *p* ≤ 0.01, η_*p*_^2^ = 0.39] (Figure 3D). No other significant effects were found for the SCL and SCR indices.

## 4. Discussion

The purpose of the present research was to examine the impact of the explicit IA manipulation on autonomic synchrony during synchronization tasks that required both motor and linguistic synchronization. For this study, an hyperscanning approach was applied to allow the recording of participants’ inter-individual autonomic responses related to the motor and linguistic synchronization tasks. For the psychophysiological signal, we also performed the analyses of the coherence indices on multiple concomitant physiological measures and a comparison of autonomic coherence’s strength for the distinct conditions and tasks proposed.

First, it was chosen to report the main statistically significant results in graphs to describe the trend of synchronization of the dyads. Secondly, some relevant and significant findings were observed: higher synchrony for HR, HRV, SCL, and SCR values in the focus compared to no focus condition during the motor synchronization task and in general more for the motor than linguistic task. Moreover, higher synchrony was also found for HR, SCL, and SCR values during the focus than no focus condition in linguistic task. These results derived from the statistical analysis applied on inter-subject coherence indices will be discussed below.

Firstly, in line with our hypotheses, we observed higher coherence in autonomic indices (namely, HR, HRV, SCL, and SCR values) during the focus on the breath condition mainly in the motor compared to the linguistic synchronization task. This finding demonstrated that physiological synchrony between two individuals while performing even basic synchronization tasks could be affected by the simple act of focusing on one’s breathing. Previous studies have shown that during dual dynamics, breath rates synchrony also occurs between the two interagents (Bachrach et al., 2015; Tschacher and Meier, 2020), however this study demonstrates that even performing a synchronized action while paying attention to one’s body has an impact on the synchronization of autonomic markers that signal a greater level of emotional engagement, such as SCL and SCR (Balconi et al., 2017), and perception of togetherness and synchrony, like HR and HRV (Vickhoff et al., 2013; Noy et al., 2015).

Secondly, by observing the statistical significance and the average coherence trends, this autonomic synchrony effect was mainly evident for the motor synchronization compared to the linguistic synchronization task and this finding confirmed the evidence observed in the context of interoception manipulation at the neural level. Indeed, the motor synchronization task was previously shown to be more sensitive to the interoceptive manipulation in previous EEG studies (Angioletti and Balconi, 2022b), and especially in former EEG hyperscanning studies (Balconi and Angioletti, 2023a). This result was explained by the connection between breathing and motor coordination, or possibly because interoceptive networks and sensorimotor regions are neuroanatomically adjacent.

In interpersonal autonomic coupling studies, a physiological synchronization of different autonomic indices is observed in both motor and linguistic synchronization tasks (Palumbo et al., 2017). For example, Noy et al. (2015), through a “mirror game” in which participants put their hands together and moved them in synchrony, showed that physiological synchrony in HR was significantly correlated with synchronized movement, subjectively reported togetherness, and high HR. Also, Vickhoff et al. (2013) found physiological synchrony in HRV during choir singing of a hymn and mantra. It is worth noticing that the brain regulates the cardiovascular correlates both before and throughout any motor action. Thus, the coherence between the dyad components for the autonomic variables may be derived by a comparable motor planning.

However, our study showed that the focus on one’s breathing increases the autonomic markers of togetherness and emotional engagement more than in the no focus on the breath condition and more in a motor synchronization rather than in a linguistic task, thus suggesting that it is specifically the attention on breathing practices (rather than similar motor planning) to have a greater impact on synchronization motor dynamics in terms of autonomic synchrony.

Thirdly, it should be noted that higher physiological synchrony was also found for HR, SCL, and SCR values during the focus than no focus condition in linguistic task. Indeed, as previously mentioned, inter-individual physiological synchrony was also identified during dyadic conditions requiring verbal or linguistic synchronization (Palumbo et al., 2017).

Differently from previous studies observing significant effect of physiological synchrony on HRV during verbal conditions (Müller and Lindenberger, 2011; Vickhoff et al., 2013), no significant findings were found for HRV for the linguistic task condition. A possible explanation could be that the feature of the linguistic synchronization task used in this study (a modified version of an alternate speech task) that is different from choir singing, independently of IA manipulation, also had an impact on the outcomes. In future studies, it could be relevant to employ a widely used linguistic and verbal synchronization condition, for example chatting or singing a hymn or in choir.

Despite the originality of this work and the reliability of inter-subject coherence indices as valid markers of physiological synchronization, when the interoceptive focus is manipulated, some caveats should be highlighted. First, the reduced number of dyads collected through a convenience sampling approach can be increased for augmenting the reliability of current findings at the inter-individual level. Indeed, for this study, no power analysis was conducted in the absence of a population that could serve as a reference sample.

In addition, although the primary goal of this study was to test the attention on the breath rather than the control on respiration, and physiological synchrony was already tested on multiple concurrent physiological measures, as suggested by Palumbo et al. (2017), future studies may consider testing synchrony even on respiratory measures (such as respiratory rate, respiration volume or compound indices such as respiratory sinus arrhythmia). Moreover, by adding an explicit social manipulation (i.e., a social frame, as done in previous studies; Balconi and Angioletti, 2023b) and a control condition (i.e., a motor and linguistic task that do not require the synchronization), future studies could test the effect of an explicit social frame and synchronization on autonomic synchrony.

Finally, a full explanation of our results in terms of positive or negative valence cannot be provided by physiological synchrony and subjective sense of synchronization alone. Thus, subsequent research could introduce some behavioral metrics (such as reaction times) and self-report measures for elucidating the social and affective components of IA manipulation on inter-individual synchronization.

To sum up, the current hyperscanning research displays how manipulating IA, which is attained by concentrating the attention on breathing, enhances the expression of interpersonal tuning in autonomic signals during two simple synchronization tasks. In particular, this work showed that the focus on one’s breathing increases the autonomic markers of togetherness and emotional engagement more in a motor synchronization rather than in a linguistic condition, suggesting that the IA manipulation effect and focus on the breath practices can have a greater impact on motor synchronization dynamics.

By examining the role of interoception in a two-person interactive social dynamic and its relation to autonomic synchrony, this study provided the first autonomic evidence in the field of social interoception that could be of interest to basic research. Moreover, these findings could be useful, for instance, for rehabilitation professionals, as they suggest that the focus on the breath during synchronized dyadic motor exercises can result in positive effects on the autonomic level and consequently this might have a beneficial effect on the effects of rehabilitation. Such latter direct impact of the present results could be explored in other future applied research in the field of motor rehabilitation.

## Data availability statement

The raw data supporting the conclusions of this article will be made available by the authors, without undue reservation.

## Ethics statement

The studies involving human participants were reviewed and approved by the Department of Psychology, Catholic University of the Sacred Heart, Milan, Italy. The patients/participants provided their written informed consent to participate in this study.

## Author contributions

MB and LA: conceptualization, methodology, and data curation. LA: software. MB: validation, formal analysis, investigation, resources, supervision, project administration, and funding acquisition. LA and RA: writing—original draft preparation. MB, RA, and LA: writing—review and editing. MB and RA: visualization. All authors contributed to the article and approved the submitted version.
